# Mutational patterns along different evolution paths of follicular lymphoma

**DOI:** 10.3389/fonc.2022.1029995

**Published:** 2022-11-10

**Authors:** Miri Michaeli, Emanuela Carlotti, Helena Hazanov, John G. Gribben, Ramit Mehr

**Affiliations:** ^1^ The Mina and Everard Goodman Faculty of Life Sciences, Bar Ilan University, Ramat Gan, Israel; ^2^ Center for Haemato-Oncology, Barts Cancer Institute – a CR-UK Centre Of Excellence, Queen Mary University of London, London, United Kingdom

**Keywords:** somatic hypermutation, high-throughput sequencing, follicular lymphoma, clonal evolution, B lymphocytes

## Abstract

Follicular lymphoma (FL) is an indolent disease, characterized by a median life expectancy of 18-20 years and by intermittent periods of relapse and remission. FL frequently transforms into the more aggressive diffuse large B cell lymphoma (t-FL). In previous studies, the analysis of immunoglobulin heavy chain variable region (IgHV) genes in sequential biopsies from the same patient revealed two different patterns of tumor clonal evolution: direct evolution, through acquisition of additional IgHV mutations over time, or divergent evolution, in which lymphoma clones from serial biopsies independently develop from a less-mutated common progenitor cell (CPC). Our goal in this study was to characterize the somatic hypermutation (SHM) patterns of IgHV genes in sequential FL samples from the same patients, and address the question of whether the mutation mechanisms (SHM targeting, DNA repair or both), or selection forces acting on the tumor clones, were different in FL samples compared to healthy control samples, or in late relapsed/transformed FL samples compared to earlier ones. Our analysis revealed differences in the distribution of mutations from each of the nucleotides when tumor and non-tumor clones were compared, while FL and transformed FL (t-FL) tumor clones displayed similar mutation distributions. Lineage tree measurements suggested that either initial clone affinity or selection thresholds were lower in FL samples compared to controls, but similar between FL and t-FL samples. Finally, we observed that both FL and t-FL tumor clones tend to accumulate larger numbers of potential N-glycosylation sites due to the introduction of new SHM. Taken together, these results suggest that transformation into t-FL, in contrast to initial FL development, is not associated with any major changes in DNA targeting or repair, or the selection threshold of the tumor clone.

## Introduction

Follicular lymphoma (FL) is the second most common non-Hodgkin lymphoma. It is an indolent disease, clinically characterized by intermittent relapses and remissions (1) with about a third of cases transforming into a more aggressive lymphoma, most commonly diffuse large B cell lymphoma (t-FL) (2–4).

Previous analysis of immunoglobulin heavy chain variable region (IgHV) genes performed on sequential biopsies from the same patient revealed at least two different patterns of clonal evolution: direct evolution through acquisition of additional somatic mutations over time, and divergent evolution, in which later FL clones come from a less-mutated common progenitor cell (CPC) (5–9), that has escaped treatment and given rise to new diverse tumors.

High-throughput sequencing (HTS) has a great advantage over classical sequencing methods in the field of immunoglobulin (Ig) gene research, as it enables us to simultaneously analyze and compare many samples at a great depth (10–19). By analyzing the qualitative and quantitative pattern of SHM we were able to understand whether changes in the mutation pathways, including the creation of U:G mismatches by the enzyme activation-induced cytidine deaminase (AID), and their correction by error-prone DNA repair mechanisms, may be responsible for some transformation events [reviewed in (20)]. More recently, lineage tree-based mutation analysis has proven to provide better mutation counts than the sequence-based analysis, allowing to count only once the mutations shared between different progeny cells and enabling us to identify reversal mutations (21, Neumann et al., Front. Immunol., *in press*). In a previous lineage tree-based analysis, performed on 40 indolent and 39 aggressive lymphomas, we showed that lymphoma trees were more branched and had longer trunks – features of higher intraclonal diversification and a longer mutational history – compared to those from controls (21). However, tumor clones exhibited similar mutation frequencies (numbers of mutations per sequence) and identical SHM motifs to those observed in not-tumor B cells. These results suggested that the observed differences were probably a consequence of the longer diversification times of lymphoma clones rather than changes in their mutation rates (numbers of mutations per sequence per cell division). FL, which is considered a less aggressive lymphoma, displayed higher intraclonal diversity than Diffuse Large B Cell Lymphoma (DLBCL) and higher numbers of recent diversification events, confirming that the most aggressive lymphoma diversifies the least as it usually has less time to diversify until it is discovered and treated.

Our goal in the present study was to characterize at a greater depth the SHM patterns of FL tumor clones from sequential relapsed/transformed samples. One of SHM outcomes is the creation of N-glycosylation sites (22). N-glycosylation sites are rare in germline (GL) sequences from healthy individuals (23), but FL clones have been proven to acquire N-glycosylation sites on the heavy or light chains of the immunoglobulin gene (24). Recently, N-glycosylation sites have been also described in some autoimmune diseases (25). Thus, N-glycosylation sites, whether in the germline or acquired, may have a critical role in the development and selection of malignant clones.

## Materials and methods

### Samples, RNA extraction, amplification and sequencing

Biopsies were obtained after written informed consent in accordance with the Declaration of Helsinki and approval from the North East London Research Committee. Three patients were included in the study: Patient no.1 (Pt1) had three samples, one t-FL and two FL; Patients no. 2 and 3 (Pt2 and Pt3) had 2 FL samples each. All tumor samples carried an IgH-VH3 rearranged major tumor clone; they were selected, and RNA extracted and amplified, as previously described (26). Briefly, 37 libraries (seven from the whole lymph node biopsies corresponding to the three patients, and the rest from flow-sorted B cell sub-populations) were prepared in the Gribben lab using JH consensus and VH3-FR1 primers (5) containing unique molecular identification (MID) tags for sample identification. Libraries were sequenced using the Roche 454 Life Sciences Genome Sequencer FLX following the manufacturer’s instructions for the Titanium series (454 Life Science, Roche).

In addition, 32 FL samples (FL-S) containing 772 sequences (21) and nine healthy germinal center (GC) samples from spleen and Peyer’s patches containing 129 sequences, previously analyzed by the Mehr lab (27, 28), were also included in the analysis and used as controls. Comparisons were performed between tumor and non-tumor clones from the three FL patients of this study (FL-HTS), and between them and the previously studied FL-S and healthy GC clones. An intra-patient analysis was also performed by comparing tumor clones from sequential samples from the same patient, collected from different anatomical sites (Pt3) or at different stages of the malignancy or treatment (Pt1 and Pt2, FL/t-FL, **Table S1**).

### Data pre-processing

Reads of the tumor clone in each sample were first processed as described (26). Briefly, data were identified by BLAST against the sequences obtained by homo/heteroduplex analysis, separated by their sample molecular identification (MID) tags, and filtered to remove reads of length ≤60 nucleotides (excluding MID tags and primers) or reads captured only once. Reads captured only twice were examined manually and included in the study only if the pyrograms showed high quality throughout. Remaining paired-end reads were assembled, annotated by SoDA (29) and aligned by ClustalW2 (30, 31) before and again after removal of insertions and/or deletions suspected to be artifacts.

In order to discard artifactual insertions and/or deletions (henceforth called indels, typically introduced during the 454 Roche sequencing) we used our program Ig-Indel-Identifier (Ig Insertion – Deletion Identifier) (32). We assumed that the two other datasets (32 FL samples and 9 GC samples), generated by using the Sanger method, did not contain any such artifacts and were therefore excluded from this initial analysis. In order to include as large a number of reads as possible in the analysis, we ran the Ig-Indel-Identifier program with the following, permissive parameter values: the minimum homopolymer tract (HPT, a stretch of identical nucleotides) length that must be checked was set to 2 nucleotides, the minimum number of sequences from the same B cell clone that must share the same indel or a low quality score point mutation for this indel or mutation to be considered legitimate was set to 1, and no exclusion of low-quality point mutations. Only unique sequences, which differ from all other sequences by one or more mutations, were kept for further analyses. A total of 2,381 unique sequences without suspected artifact indels were used for further analyses (**Table 1**).

**Table 1:** Numbers of clones, unique sequences and mutations in tumor and non-tumor clones in each sample, in each patient.

Patient	Sample*	Clones**	No. of clones	No. of unique sequences***	No. of mutations****			
					Total	Min	Max	Med
Pt1	1	T	1	166	263	NA	NA	NA
		NT	21	51	548	3	44	34
	2	T	1	141	229	NA	NA	NA
		NT	11	22	373	11	42	37
	3	T	1	235	368	NA	NA	NA
		NT	34	99	731	3	55	19.5
Pt2	1	T	2	108	257	33	224	128.5
		NT	9	13	279	27	39	30
	2	T	1	407	622	NA	NA	NA
		NT	7	23	206	4	44	30
Pt3	1	T	2	345	438	209	229	219
		NT	13	42	443	5	49	33
	2	T	2	721	813	406	407	406.5
		NT	4	8	175	33	51	45.5

*Samples are numbered chronologically. **T, tumor clones; NT, non-tumor clones; clones are defined as described in the methods. ***Unique sequences are sequences that differ from all other sequences by one or more mutations. ****The numbers of mutations were calculated from the lineage trees. This way, we counted each mutation only once if it happened earlier in the clone. We present the total, minimum (Min), maximum (Max) and median (Med) numbers of mutations. The V(D)J gene segment combinations detected in all samples analyzed on the 454 Roche sequencer are given in **Supplementary Table S2**. NA, Not Applicable.

### Germline VDJ segment identification and assignment into clones

Clonally related sequences were defined as reads having identical V,D, and J segments according to SoDA (29); if there were two or more clones with the same V, D and J segments in a sample (as shown in **Table S2**), only sequence groups with highly homologous sequences of complementarity determining region 3 (CDR3) were considered as clones, as confirmed by visual examination of the alignments after sequences with the same V, D and J segments were aligned using ClustalW2 (30, 31). Since libraries were prepared using VH3-FR1 primers, VH3 genes from non-tumor B cells (NT) were also amplified and sequenced. Tumor-related reads (T) were defined as the reads identical to the dominant sequence plus those sequences that, based on the SHM pattern of IgH-VH, were clonally related to the dominant tumor sequence. Clones with the same V(D)J segments but with completely different CDR3 sequences (no shared nucleotides) and no shared mutations elsewhere were considered as non-tumor clones. **Table 1** shows the numbers of clones, unique sequences and mutations in the tumor and non-tumor clones in each sample, in each patient.

### Mutational analyses

#### Ig lineage tree analyses

Clonally-related Ig gene sequences were used to generate lineage trees (**Figure S1**) using our program IgTree^©^ (33), as previously described (21, 34). The lineage trees were then measured using our program MTree^©^ (35, 36). In a previous study, a thorough statistical analysis performed on simulated data has established the quantitative relationships between lineage tree characteristics and the parameters characterizing affinity maturation dynamics (proliferation, differentiation and mutation rates, initial affinity of the Ig to the antigen, and selection thresholds); seven specific characteristics (the minimum root to leaf path length, the average distance from a leaf to the first split node/fork, the average outgoing degree, that is the average number of branches coming out of any node, the root’s outgoing degree, the minimum distance between adjacent split nodes/forks, the length of the tree’s trunk and the minimum distance from the root to any split node/fork) were the most informative (37). The comparison between lineage tree characteristics from different patients or between different datasets was done using the non-parametric Mann-Whitney U-test, as these characteristics are not always normally distributed. To correct for multiple comparisons, we used the false detection rate (FDR) correction method (38).

#### Mutation distributions

The analysis of mutation distributions (or mutation spectra, together with targeting motif analysis described in the next section) enables us to characterize the SHM mechanisms operating in the B cell clones. The numbers of mutations from A, C, G, and T were counted for each sample and expressed as percentages of the total number of mutations detected in each sample. When different samples were compared, the *expected* numbers of mutations from A, C, G, and T in each sample were calculated as the observed number of mutations from either A, C, G, or T in that sample, multiplied by the total number of mutations in that sample and divided by the total number of mutations of the two samples. A χ² analysis was then performed on all mutation numbers, comparing between the sets of observed and expected mutation numbers. In addition, the ratios of the percentages of transition and transversion mutation (from the total number of mutations for each group) were examined in each sample. A χ² test was performed to compare the tumor and non-tumor transition and transversion percentage ratios in all patients and samples.

#### SHM targeting motif analysis

It is established that SHMs occur at higher frequency in specific sequence motifs (39, 40). Identification of SHM targeting motifs around mutated positions was performed as described (41) to further examine the mechanism of SHM. This analysis was based on a previous published work by Spencer and Dunn-Walters (42). Briefly, the base composition at positions flanking a mutation (three nucleotides on either side) was determined and then, for each nucleotide, a χ² test was performed to check whether the frequency of each type of mutation was statistically significant compared with the background frequency observed in the germline (GL) sequence. The F-test was used to compare the base compositions surrounding the mutations from different datasets.

#### N-glycosylation analysis

We first analyzed the potential glycosylation sites in the GL sequences, and then the acquired glycosylation sites (AGS) introduced by SHM in the mutated sequences. The N-glycosylation motif included in the analysis was Asn–X–Ser/Thr, where X is any amino acid except Pro, Asp or Glu. To analyze the potential glycosylation sites in the GLs, we counted the number of occurrences of the full motif, and – separately – the occurrences of motifs which differ by one nucleotide from the full motif. We compared the two numbers between tumor vs. non-tumor clones, in order to establish whether N-glycosylation may have affected clonal dynamics. The χ² test was used to compare the groups; in clones with less than five GLs and more than one glycosylation site, for which the χ² test did not apply, a likelihood ratio was used instead. In the analysis of AGS, we compared the number of clones with AGS and the number of AGS per clone between tumor and non-tumor clones in each sample and patient.

## Results

### SHM mechanisms in FL clones may be different from those in normal clones

**Figure 1:**
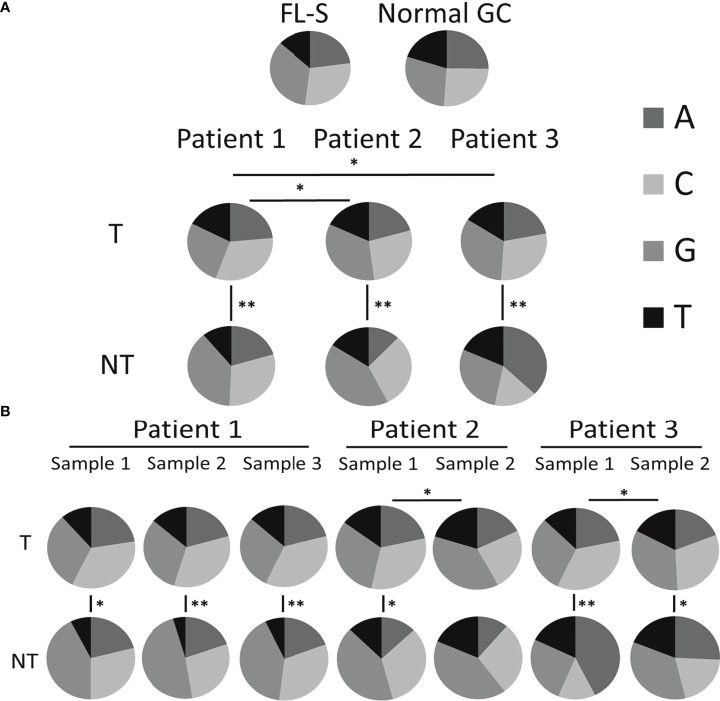
Mutation spectra (distribution among nucleotides). The number of mutations from each nucleotide, presented as a fraction out of the total number of mutations, for **(A)** tumor and non-tumor clones in each patient analyzed (also including the reference FL samples from a previous study and control GC samples) and **(B)** for tumor and non-tumor clones in each sample analyzed. Significant differences are indicated with lines between pie-charts (*p-value < 0.05; **p-value < 0.0005). Data regarding the numbers of mutations from each nucleotide and p-values of the comparisons between the different groups are shown in **Tables S3, S4 and Tables S5, S6**, respectively.

Our hypothesis is that changes in mutational mechanisms, including AID targeting and the subsequent error-prone correction by DNA repair mechanisms, may be responsible for some transformation events. Several lines of evidence in this study show that in some cases a change in SHM mechanisms may have occurred in the tumor clone between biopsies. First, we observed that the mutation spectra of tumor clones were different from that of non-tumor clones at both the patient and the sample levels; the only exception was sample number 2 of Pt2, displaying similar mutation spectra in both tumor and non-tumor clones. The mutation spectra of tumor clones from all patients were similar (**Figure 1A**), while the non-tumor clones from all patients presented unusual and highly variable mutation spectra, mostly in the non-tumor clones of sample 2 from Pt3 and samples 1, 2 and 3 from Pt1; this variability may be due, in part, to the relatively low numbers of unique sequences detected in these NT clones, combined with the intrinsic randomness of SHM. Tumor clones from all three Pt1 samples were similar in their mutation spectra (**Figure 1B**), suggesting that the mutation mechanisms did not change over the elapsed time. On the other hand, mutations from G to any other nucleotide were found to be more frequent than mutations from C to any other nucleotide in tumor clones of the second (later) biopsies of both patients 2 and 3 than in the earlier samples, implying that some mutational mechanisms (possibly the targeting) may have changed between the two consecutive biopsies of each of these patients.

**Figure 2:**
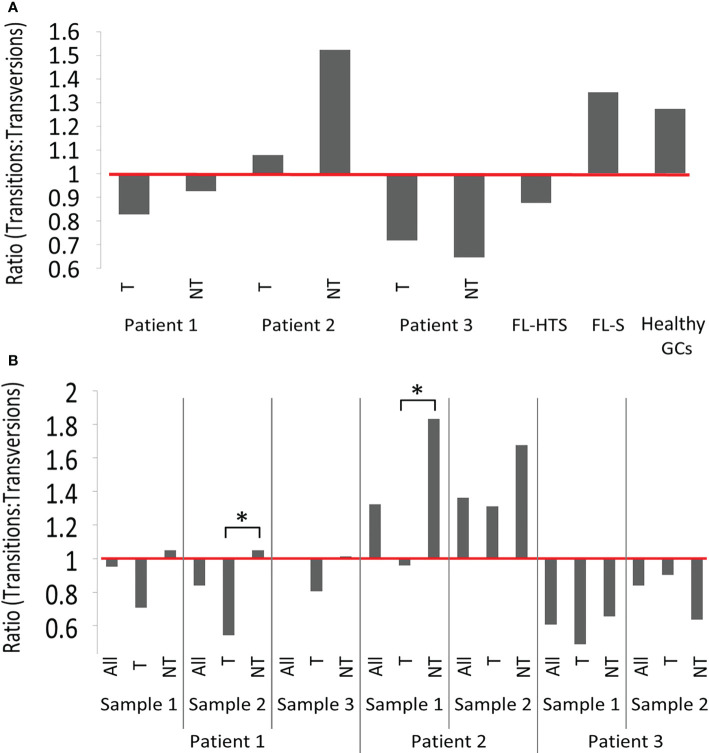
Transition-transversion mutation ratios. **(A)** In tumor and non-tumor clones per patient analyzed and **(B)** In tumor and non-tumor clones per sample. Additional information regarding the numbers of transition and transversion mutations in tumor and non-tumor clones of FL patients, and in healthy GCs are provided in the **Tables S7 and S8**. According to the χ² test, no significant differences were observed between tumor and non-tumor clones across different patients. Significant differences were found when tumor and non-tumor clones from sample 2 from patient 1 and sample 1 from patient 2 (*p-value < 0.005) were compared. (See also **Tables S7 and S8**).

Second, the transition-transversion mutation ratios greatly varied between patients and samples. Transition mutations are point mutations that replaces a purine nucleotide by another purine or a pyrimidine by another pyrimidine, while a transversion is a replacement of a purine by a pyrimidine or vice-versa. A transition-transversion ratio larger than 1 means that there were more transition mutations than transversion, and vice versa for a ratio smaller than 1; in normal B cell clones, this ratio is larger than 1, as in the healthy control GCs used in this study and various others [e.g. (43)]. Pt2 had transition-transversion ratios larger than 1 in all clones in both samples except the tumor clone in sample 1, while Pt3 presented transition-transversion ratios smaller than 1 (**Figure 2**) in all clones. In non-tumor clones from Pt1 the ratios were ~1:1, while they were <1 in the tumor clones. Note that, when samples were combined together to look at a more complete picture for each clone, the lineage tree structure of each clone – and hence the characteristics of some mutations – may have changed. Significant differences between tumor and non-tumor clones were found only in the second FL sample of Pt1 and in the first FL sample of Pt2. These results raise the question of whether DNA repair mechanisms are altered with transformation in these FL cases. According to a previously published paper, the polymerase Rev1 may promote transversions at C:G pairs, while the low-fidelity polymerase θ can introduce both transitions and transversions at abasic sites (44). Alternatively, BCR-based selection may be impaired, if it operates at all, in FL clones, as in other lymphomas (21, 43).

**Figure 3:**
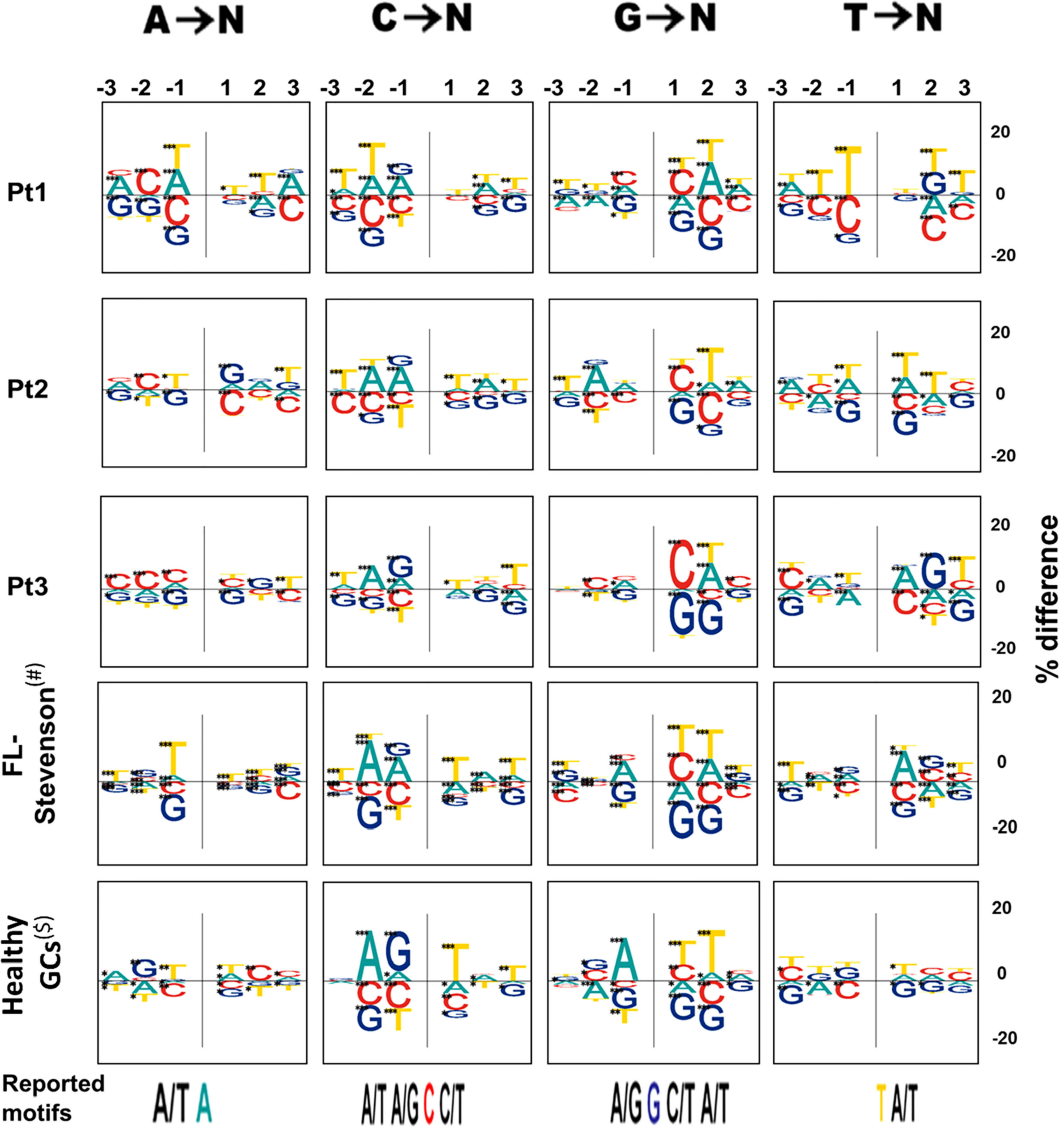
Mutation targeting motifs. On the top we show the 3 nucleotides examined upstream and downstream, for each mutated nucleotide, denoted as -1, -2, and -3, for the 3 positions flanking the mutation upstream and 1, 2, and 3 for those flanking the mutation downstream. The positive and negative sides of the Y axis denote excess or paucity of the indicated nucleotide in that position, respectively. The size of each letter is given by the “% difference”, calculated as percentage of each base at each position flanking a particular mutation, minus the percentage composition of the GL sequence at that position. Asterisks represent levels of significance (*p-value < 0.05; **p-value < 0.005; ***p-value < 0.0005). Previously reported motifs are shown at the bottom of the figure; the mutated nucleotide is colored and the flanking nucleotides are shown for reported positions.

Third, all FL-HTS samples presented different mutation targeting motifs for mutations from G relative to the reported motif (**Figure 3**). The healthy GCs samples, used as controls, presented the reported motif but not the new motifs, supporting the hypothesis that a possible change in the SHM mechanism occurred in at least one FL case. When we examined how many positions in the motif for each mutation contained significant differences between the tumor and non-tumor clones in each patient, we observed that T and NT clones from Pt1 were the most similar in terms of motif usage. In addition, consecutive tumor samples from the same patient did not have statistically significantly different mutation targeting motifs (**Tables S9-S12**). Taken together, the differences presented above suggest that SHM mechanisms (targeting, DNA repair or both) may be different in FL compared to normal B cell clones.

### Tumor clones acquire more new potential N-glycosylation sites than non-tumor clones

It is known that only a minority of GL V segments and normally-developed memory B cell V region genes contain potential N-glycosylation sites (PGS) (23). In contrast, human B-cell malignancies, and FL in particular, are characterized by an extremely variable incidence of acquired N-glycosylation sites (AGS) in their Ig variable region sequences (23, 24, 45–47). Hence, we examined the potential and acquired N-glycosylation motifs in GL sequences and in tumor and non-tumor clones in order to determine whether potential N-glycosylation sites are more frequent in FL and t-FL than in healthy B cell clones.

**Figure 4:**
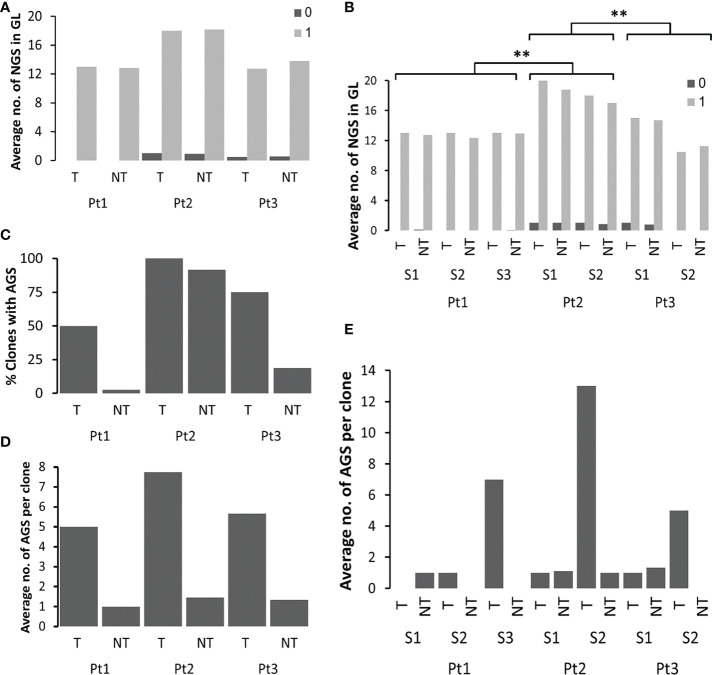
Analysis of PGS and AGS in FL samples. **(A)** Average numbers of existing potential PGS (0) and number of motifs that are only one mutation away from becoming an AGS (1) in clonal GL sequences, in tumor and non-tumor clones in each patient, and **(B)** in each sample in each patient. In case there was only one clonal GL sequence, the average number is the actual number. **(C)** Percentages of clones with AGS out of all clones in each patient. **(D)** Average numbers of AGS in tumor and non-tumor clones from each patient, and **(E)** from each sample in each patient. **p-value < 0.005.

As shown in **Figure 4A**, Pt1 had no GL sequences with existing N-glycosylation motifs, while Pt2 and Pt3 had no more than one GL sequence each that contained such motifs. However, in Pt1 and Pt3 there were, on average, 12 motifs that were only one mutation away from becoming a potential AGS. In all patients, these average numbers were similar both in tumor and non-tumor clones. After examination of the tumor and non-tumor clones from each sample (**Figure 4B**), we observed similar average numbers of PGS in clonal GL sequences, with motifs that were only one mutation away from a potential AGS in both tumor and non-tumor samples of Pt1. Pt2 presented the highest average numbers in both tumor and non-tumor GLs samples (p-value < 0.005 for both patients 1 and 3 compared to Pt2, **Table S13**).

The percentages of clones that acquired at least one new potential glycosylation site were calculated and found significantly higher in Pt2 as compared to other patients, in both tumor and non-tumor clones. Moreover, all tumor clones and 92% of non-tumor clones of Pt2 acquired at least one AGS. This is not surprising, as Pt2 had the highest percentages of clonal GL sequences with motifs that were only one mutation away from AGS. In patients 1 and 3 there were more tumor clones than non-tumor clones that acquired AGS (**Figure 4C**). Although the average numbers of motifs that are only one mutation away from becoming a potential AGS were similar between tumor and non-tumor clones in all patients (**Figure 4A**), there were more AGS per clone in tumor clones than in non-tumor clones in each patient (**Figure 4D**). Interestingly, the highest numbers of AGS per clone were found in the latter samples in each patient (**Figure 4E**). AGS in all tumor clones were present along the sequence, ranging from CDR1 to CDR3. In both Pt2 and Pt3, the highest numbers of AGS in the later tumors correlated with the highest number of mutations in the later tumors (**Table 1**). However, in Pt1, the numbers of SHMs observed in the third sample was not significantly higher than that detected in the two former samples, while the number of AGS in the third sample was much higher than the rest, implying that in addition to the influence of the number of SHMs on the number of AGS (22), there might be additional selection for N-glycosylated sequences in FL cases (or impairment of selection against them).

### FL tumor clones display more branched lineage trees and may have had lower initial affinities and selection thresholds than non-tumor and healthy GC clones

**Figure 5:**
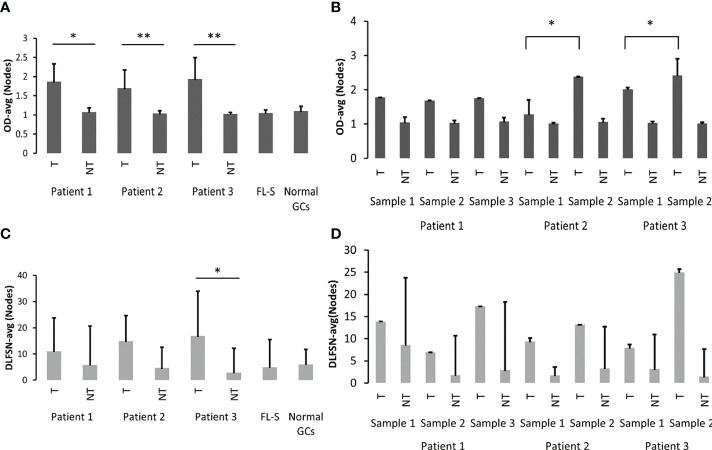
Lineage tree topologies. **(A)** Outgoing degree (OD-avg) of tumor and non-tumor clones of the three FL-HTS patients, FL-S and healthy GC samples. **(B)** OD-avg of tumor and non-tumor clones in each sample from all three FL-HTS patients. **(C)** The average distance from a leaf to the first split node/fork (DLFSN-avg) of tumor and non-tumor clones of the three FL-HTS patients, FL-S and healthy GC samples. **(D)** DLFSN-avg of tumor and non-tumor clones in each sample in each of the three FL-HTS patients. *p-value < 0.05; **p-value < 0.005.

In order to further quantify the differences between the dynamics of SHM and antigen-driven selection in healthy GCs, FL-S and FL-HTS, we performed a quantitative analysis of lineage tree topologies, using our program MTree^©^ (35, 36). Tumor clones from the three FL-HTS patient samples presented significantly larger average outgoing degree (OD-avg, that is, number of children per node), which is a branching measure, when compared to the non-tumor clones detected with HTS (**Figure 5A**). Non-tumor clones presented values around 1, indicating that most non-tumor trees were not highly branched. According to simulations (37), when trees are not highly branched, it suggests that either the initial affinity of the clone’s B-cell receptor to the driving antigen was not very high, or that antigen-driven selection was rather stringent. As in the present study we analyzed fewer sequences than in the simulations, many of the non-tumor clones contained only one sequence and (creating “sticks” rather than branched trees), although some of those may have been part of larger, branched clones that were undetected. However, we believe that this could not account for the large, significant differences in the degree of branching between tumor and non-tumor clones, as all our other studies show that OD-avg is always close to 1 in non-tumor clones (Mehr lab, unpublished data). Furthermore, tumor clones from the later biopsies of patients 2 and 3 showed significantly larger OD-avg values than those of tumor clones from the earlier biopsy in each case (**Figure 5B**). This may imply that the later tumors are more diversified than the earlier tumor clones, possibly due to weakening of the selection forces operating on the tumor clone with time.

We also observed differences in tree length measures: trunk length, minimum root to leaf path length (PL-min) and minimum distance from the root to any split node/fork (DRSN-min) (37). However, PL-min and DRSN-min mostly include the trunk length, which in turn includes the clone’s history of mutations, some of which may have been acquired before the transformation event into FL (or t-FL). Thus, in **Figure 5** we only show the average distance from a leaf back to the first split node/fork (DLFSN-avg), that is, the paths to leaves without the trunks, which were found in simulations to be correlated with lower initial affinity and selection thresholds (37). Tumor clones presented larger DLFSN-avg values than non-tumor clones, which supports the suggestion that transformation (and possibly also relapse) decreases the sensitivity of the clone to selection. This result was significant only in Pt3; although the same trend also appeared in patients 1 and 2 (**Figures 5C, D**), it was not statistically significant; this could stem from the low number of non-tumor sequences compared with tumor sequences. Overall, the larger branching and length tree measurements presented by tumor clones from the three FL-HTS samples indicate larger trees, and thus more diversification than those in healthy subject GCs.

## Discussion

Our goal in this study was to characterize the clonal evolution and SHM mechanisms of FL tumor clones, across sequential LN biopsies from the same patient (26). We observed large, highly branched lineage trees with long trunks that, together with the mutation patterns, clearly support the GC origin ascribed to FL. FL tumor clones presented more branched trees than healthy GC samples and even non-tumor clones in the same patients, with lineage tree topological measures indicative of lower initial affinities and/or selection thresholds. Moreover, these measures were similar in FL and t-FL samples from Pt1, but increased between biopsies of patients 2 and 3. Indeed, it has previously been shown that antigen-driven selection may persist after transformation and participate in diversification and progression (48–50).

We identified both direct and divergent clonal evolution patterns in the studied samples, and this was supported by mutation analyses. The similar mutation patterns in the FL/t-FL tumor clones of Pt1 may fit the hypothesis of direct evolution, while the different mutation patterns in consecutive biopsies from patients 2 and 3 may point at the existence of a CPC, different descendant clones of which were sampled in each biopsy. Moreover, the similar tree topological properties of the FL and t-FL samples in Pt1 were consistent with direct evolution, while different and, in particular, increased tree measures (that suggest a possible decrease in selection) between two consecutive biopsies in pt2 and in pt3 are also in line with the existence of a CPC.

Previous studies have shown that the analysis of DNA motifs around mutated nucleotides in the Ig genes can reveal many aspects concerning the targeting of SHM mechanism induced by AID to conserved sequence motifs (39, 40, 42, 51–57), while the analysis of mutation spectra can reveal changes in repair mechanisms (58, 59). We thus analyzed the mutation characteristics, including targeting motifs, of the seven sequential FL samples collected from the three patients, in search for evidence of such changes. Mutation distributions of tumor and non-tumor clones were different, while FL and t-FL tumor clones had similar mutation distributions. This may imply that there was no change in the SHM mechanism between t-FL and FL tumors, and that the latter transformation event did not affect these mutations. In addition, as samples from Pt1 were taken after different treatments, we may speculate that the observed SHM patterns were intrinsic to FL B cells and were not affected by the therapy. In contrast, tumor clones from consecutive samples of patients 2 and 3 differed in their mutation frequencies, suggesting that either the SHM mechanisms have changed, or there was a decrease in the tumor cell sensitivity to selection (9). Compared to a recently published analysis (60), our analysis of mutations is, on one hand, more precise, as it is done on lineage trees so that each mutation is defined relative to the closest known or deduced ancestor (Neumann et al., Front. Immunol., *in press*); and, on the other hand, it was limited to IgH coding regions, so we have no findings on non-Ig regions.

The biased frequencies of mutations from G over C we observed in FL may suggest that there was a bias for generating these mutations on only one strand during the second phase of SHM. Moreover, an elevated number of mutations from G was ascribed to DNA mismatch repair protein MutS homolog 2 (MSH2), uracil-DNA glycosylase (UNG) or DNA repair protein REV1 deficiency (61). This mutation pattern also suggests the possible intervention of a reverse transcription step in fixing the pattern in DNA (62). Furthermore, the difference in transition-transversion mutation ratios between samples raised the question of whether DNA repair mechanisms are altered in FL. Healthy replication over abasic sites after U removal by base excision repair (BER) followed by UNG can lead to G/C targeted transversion mutations (62). Thus, UNG overexpression or enhanced activity may cause transition-transversion ratios smaller than 1 in FL. Thus, SHM mechanisms in FL have to be more thoroughly examined by gene expression or proteomics for detecting enzyme expression levels.

Finally, the number of AGS was higher in tumor clones than in non-tumor clones in all FL-HTS samples, implying it is likely that AGS have some role in FL development, at least in the initial stage, as was previously suggested (47). In addition, because the later samples from all patients had the highest numbers of AGS per clone, we may conclude that the tumors accumulate AGS over time. This fits with the model of a B cell tumor population entrapped in the germinal center that keep undergoing SHM, with selection against AGS – and possibly other potentially harmful mutations – being impaired in the tumor B cells.

To summarize the mutation analyses, we found differences in the mutation distributions from each of the nucleotides, in initial clone affinity and in selection thresholds between tumor and non-tumor clones, but no differences between FL and t-FL clones. Additionally, we observed that tumor clones tend to accumulate larger numbers of potential N-glycosylation sites due to SHM. Taken together, these results suggest that transformation from FL into t-FL, in contrast to the initial transformation to FL, is not characterized by any major changes in DNA repair mechanisms, SHM, or shape of lineage trees, although the possibility of subtle changes in enzyme expression or activity should still be investigated. On the other hand, selection – at least against accumulation of AGS – seems to be impaired in FL and t-FL. This study also shows that even a few samples can provide many interesting insights, provided that these samples contain sufficient numbers of sequences and mutations.

## Data availability statement

The datasets presented in this study can be found in online repositories. The names of the repository/repositories and accession number(s) can be found below: GenBank, OP426445-OP428638.

## Ethics statement

The studies involving human participants were reviewed and approved by The North East London Research Committee. The patients/participants provided their written informed consent to participate in this study.

## Author contributions

MM developed some of the computational tools, performed all the computational analyses and wrote the manuscript, which was part of her PhD thesis in Bar-Ilan University. EC performed all the experiments and wrote the manuscript. HH developed some of the computational tools and helped with the analysis. JG supervised the experimental research. RM designed and supervised the research and wrote the manuscript. All authors contributed to the article and approved the submitted version.

## Funding

This study was supported by US-Israel Binational Science Foundation (BSF) grant number 20130432 and Israel Science Foundation grant number 270/09 (to RM). MM and HH were supported by Bar-Ilan University President’s PhD Scholarships.

## Acknowledgments

The authors are indebted to Dr. Meirav Kedmi for critical reading of an early version of the manuscript, and to Hadas Neuman for help in manuscript preparation.

## Conflict of interest

The authors declare that the research was conducted in the absence of any commercial or financial relationships that could be construed as a potential conflict of interest.

## Publisher’s note

All claims expressed in this article are solely those of the authors and do not necessarily represent those of their affiliated organizations, or those of the publisher, the editors and the reviewers. Any product that may be evaluated in this article, or claim that may be made by its manufacturer, is not guaranteed or endorsed by the publisher.
