# Supplementary material for: Mutational patterns along different evolution paths of follicular lymphoma
Source: Front Oncol. 2022 Nov 10;12:1029995. doi: 10.3389/fonc.2022.1029995 (PMC9686334; doi:10.3389/fonc.2022.1029995)

<u>Supplementary figure 1 – Lineage Trees.</u> A. Lineage trees from three representative non-dominant (NT) clones, one from each patient, chosen randomly. B. Dominant clones (T) from all 7 samples; in cases where two large clones were identified in a sample, we show the largest (TL). Patient and sample numbers are indicated on the figures, as are the V,D and J segments. Gray nodes represent the deduced pre-mutation, rearranged IgHV gene sequence, white nodes – deduced split nodes, and black nodes – sequences observed in the analysis. A number next to an edge is the number of mutations that edge represents; if there is no number, the edge represents one mutation.

A.

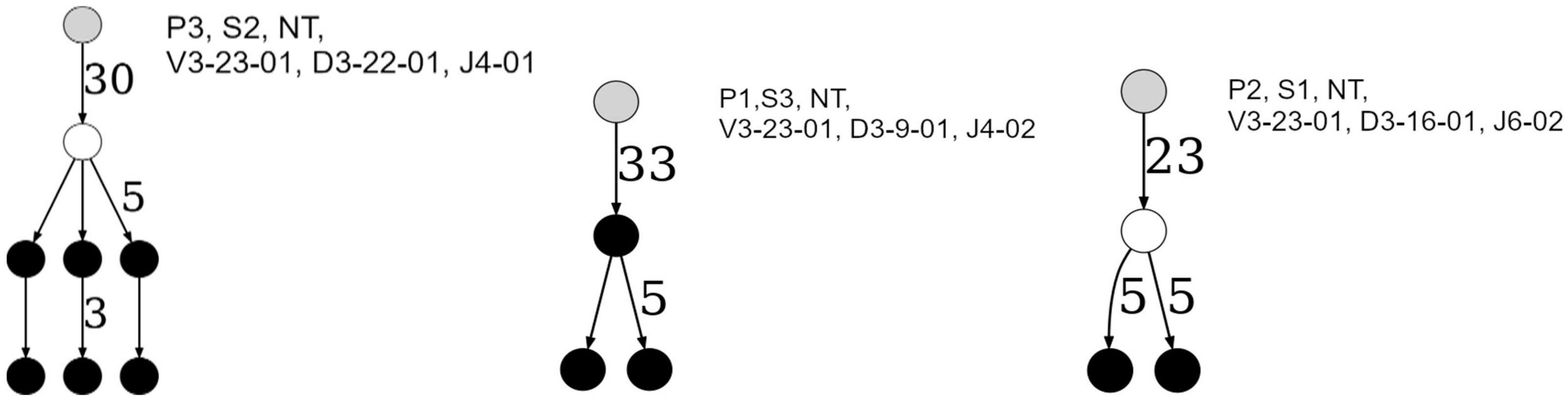

B.

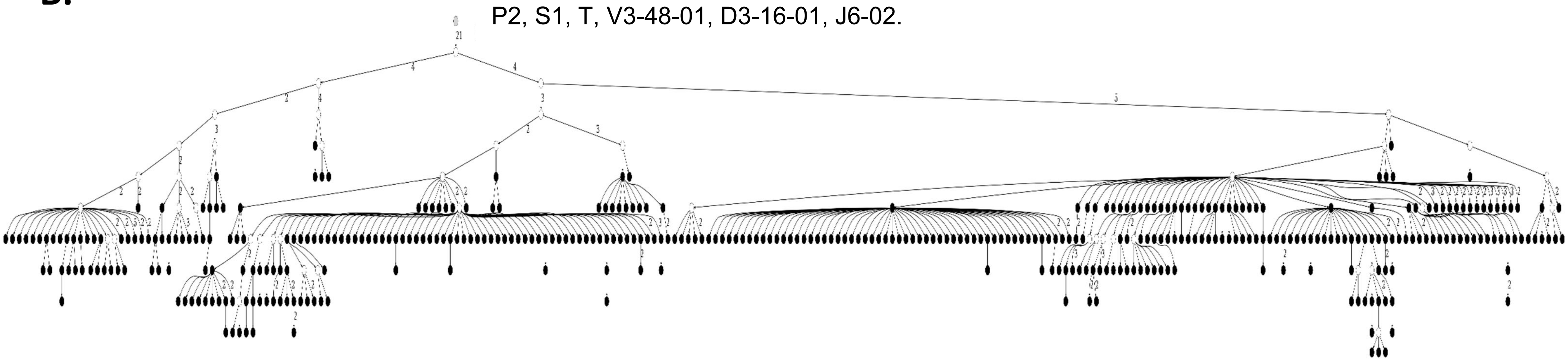

P1, S1, T, V3-23-01, D3-16-01, J1-01

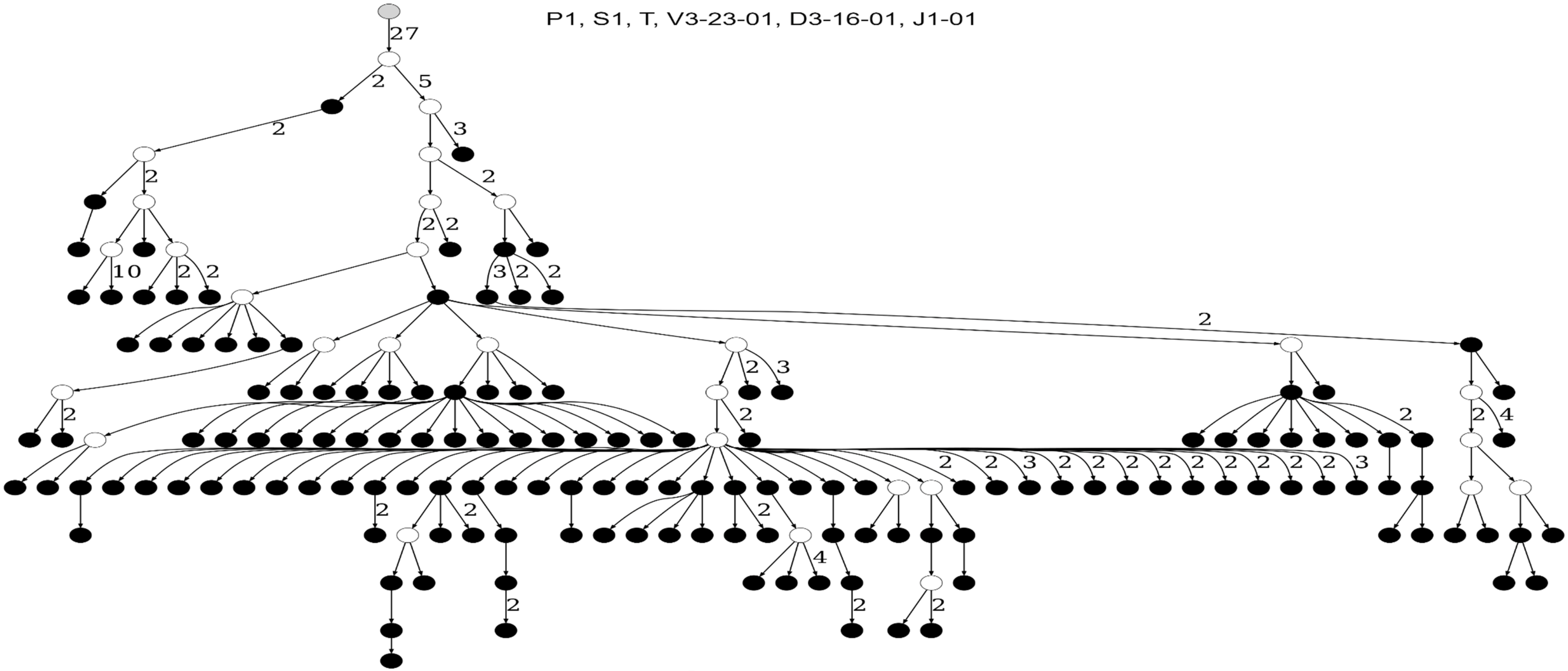

P1, S2, T, V3-23-01, D3-16-01, J1-01

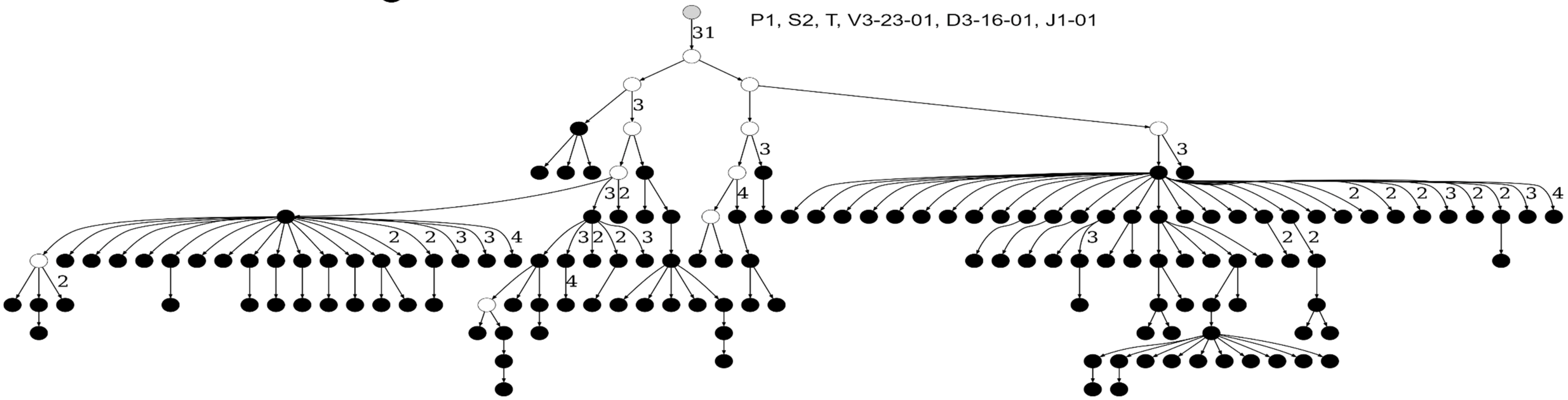

P1, S3, T, V3-23-01, D3-16-01, J1-01

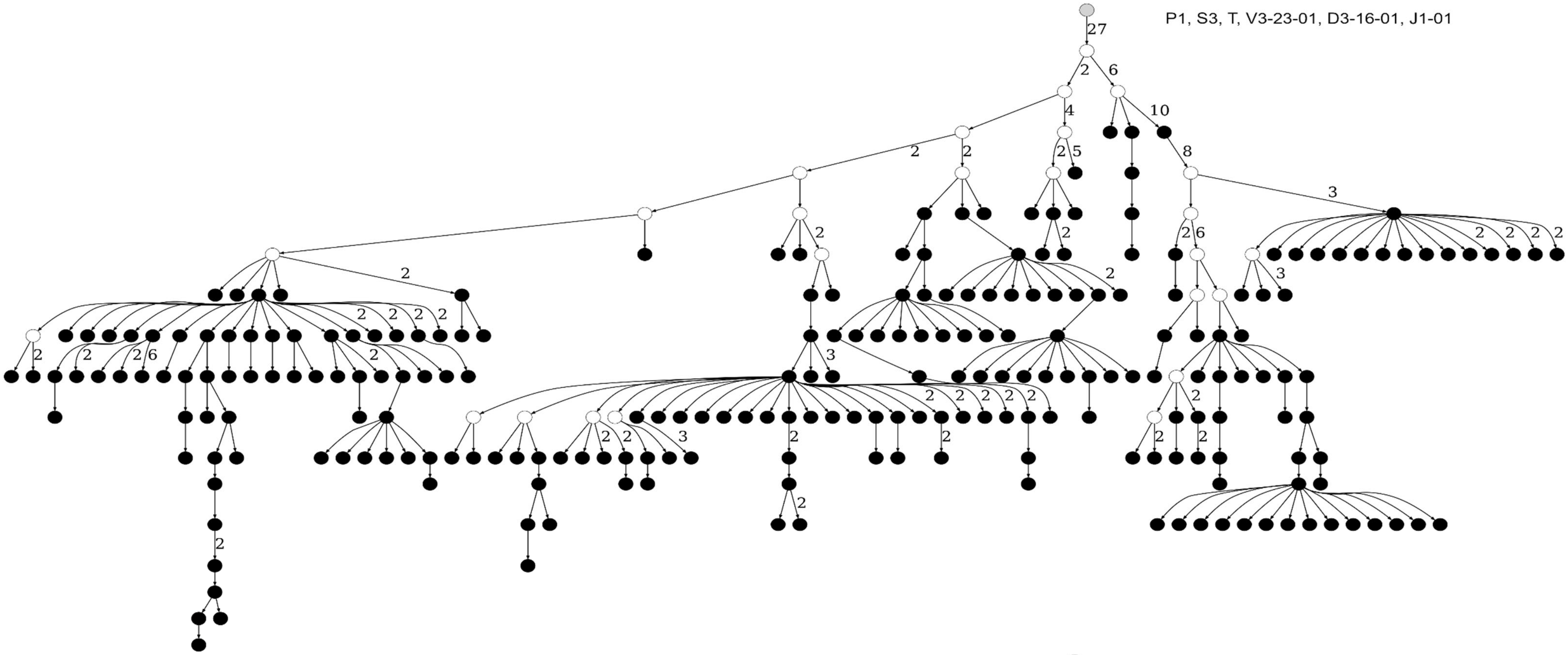

P2, S2, TL, V3-48-01, D3-16-01, J6-02

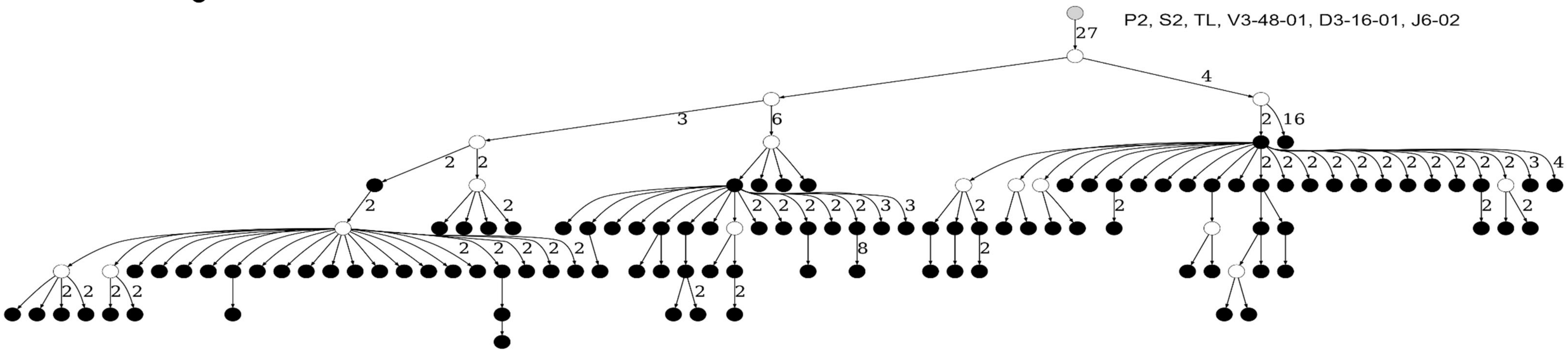

P3, S1, TL, V3-23-01, D unknown, J2-01.

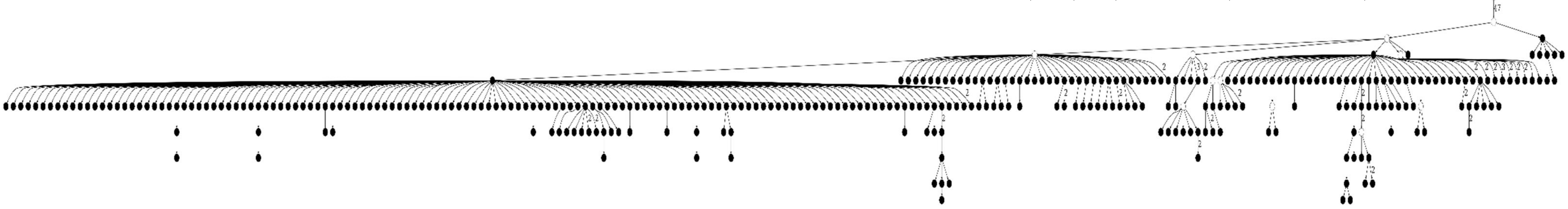

P3, S2, TL, V3-23-01, D3-10-01, J4-01.

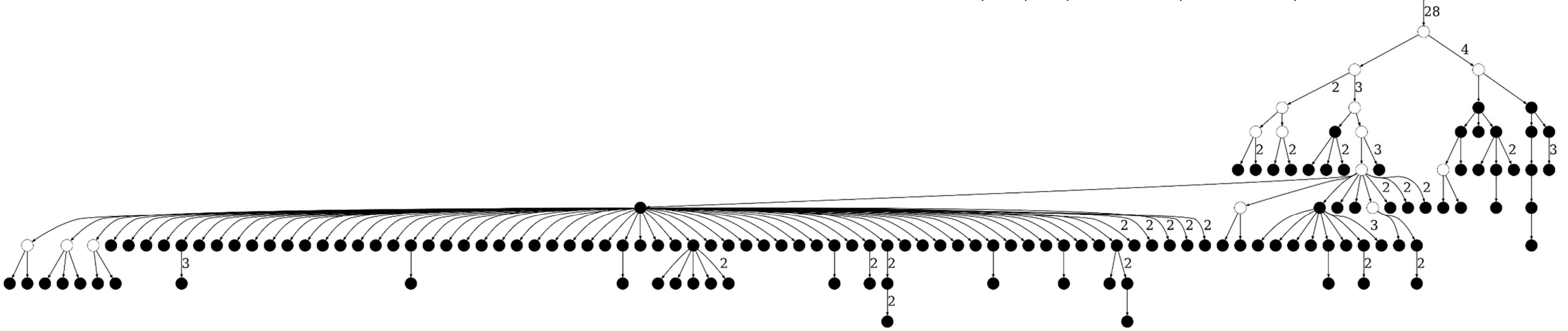

Supplement: Supplementary file 1 [file DataSheet_1.pdf]
