# Supplementary material for: Mutational patterns along different evolution paths of follicular lymphoma
Source: Front Oncol. 2022 Nov 10;12:1029995. doi: 10.3389/fonc.2022.1029995 (PMC9686334; doi:10.3389/fonc.2022.1029995)
Supplement: Supplementary file 2 [file DataSheet_2.pdf]

Supplementary Tables

<u>Table S1</u>: Summary of patient and sample data

Patient No.	Sample No.	Tumor*	Site of biopsy	Remarks**	Lines of therapy***
1	3	t-FL	Left axilla	R5	5
	2	FL	Left axilla	R6	2
	1	FL	Left axilla	P	0
2	2	FL	Left inguinal biopsy	P	5
	1	FL	Left inguinal biopsy	P	2
3	2	FL	Right axilla	WW	0
	1	FL	Right femoral node	WW	0

*FL = Follicular lymphoma; t-FL = transformed Follicular lymphoma.
**R = relapse; P = progression; WW = no treatment received before biopsy – just watch and wait.
***Number of lines of therapy received before or between biopsies.

<u>Table S2 is on the next two pages.</u>

<u>Table S3</u>: Number of mutations from each nucleotide in tumor and non-tumor clones in each patient

Sample		Number of mutations			
		from A	from C	from G	from T
Pt1	tumor	223	304	259	164
	non-tumor	209	312	395	112
Pt2	tumor	214	286	355	187
	non-tumor	47	121	164	62
Pt3	tumor	297	396	452	213
	non-tumor	219	93	168	107
FL-S		601	765	917	348
Normal GCs		172	177	197	137

<u>Table S4</u>: Number of mutations from each nucleotide in tumor and non-tumor clones in each sample, in each patient

Patient and sample			Number of mutations			
			from A	from C	from G	from T
Pt1	Sample 3	T	59	91	83	30
		NT	115	159	233	41
	Sample 2	T	47	78	73	31
		NT	73	104	179	17
	Sample 1	T	77	132	110	49
		NT	141	238	301	51
Pt2	Sample 2	T	55	82	82	38
		NT	36	92	116	35
	Sample 1	T	111	152	234	125
		NT	23	59	86	38
Pt3	Sample 2	T	95	154	134	55
		NT	189	60	115	79
	Sample 1	T	154	246	273	140
		NT	45	36	61	33

Table S2: V(D)J gene combinations detected in all clones from the FL-HTS samples

Patient	Sample	Type	V__D__J gene combinations*
Pt1	3	T	3-23-01__3-16-01__1-01
		NT	3-20-01__3-16-01__1-01
			3-23-01__0-IR-01__1-01 (1)
			3-23-01__0-IR-01__1-01 (2)
			3-23-01__0-IR-01C__1-01
			3-23-01__0-IR-01C__4-02
			3-23-01__1-IR1-01__1-01 (1)
			3-23-01__1-IR1-01__1-01 (2)
			3-23-01__2-21-01__1-01
			3-23-01__2-OF15-2__1-01 (1)
			3-23-01__2-OF15-2__1-01 (2)
			3-23-01__3-9-01__4-02 (1)
			3-23-01__3-9-01__4-02 (2)
			3-23-01__3-10-01__1-01
			3-23-01__3-16-01__3-02
			3-23-01__3-16-01__4-01
			3-23-01__3-16-01__4-02
			3-23-01__4-17-01__4-02
			3-23-01__5-5-01__1-01
			3-23-01__7-27-01__1-01
			3-30-02__3-16-01__1-01
			3-33-01__3-16-01__1-01
	2	T	3-23-01__3-16-01__1-01
		NT	3-23-01__0-IR-01__1-01
			3-23-01__1-14-01__1-01
			3-23-01__2-2-01__1-01
			3-23-01__2-OF15-2__1-01 (1)
			3-23-01__2-OF15-2__1-01 (2)
			3-23-01__3-3-01__1-01
			3-23-01__3-9-01__4-02
			3-23-01__3-16-01__3-02
			3-23-01__3-16-01__4-01
			3-23-01__3-16-01__4-02
			3-23-01__7-27-01__1-01
	1	T	3-23-01__3-16-01__1-01
		NT	3-20-01__3-16-01__1-01
			3-23-01__0-IR-01__1-01
			3-23-01__0-IR-01__4-02
			3-23-01__0-IR-01C__1-01
			3-23-01__0-IR-01C__5-02
			3-23-01__1-1-01__1-01
			3-23-01__1-7-01__1-01
			3-23-01__1-14-01__1-01
			3-23-01__1-26-01__4-02
			3-23-01__1-IR1-01__1-01 (1)
			3-23-01__1-IR1-01__1-01 (2)
			3-23-01__1-IR1-01__1-01 (3)
			3-23-01__1-IR1-01__4-02 (1)
			3-23-01__1-IR1-01__4-02 (2)
			3-23-01__2-2-01__1-01
			3-23-01__2-2-01__4-02
			3-23-01__2-IR2-01__4-02
			3-23-01__2-OF15-2__1-01 (1)
			3-23-01__2-OF15-2__1-01 (2)
			3-23-01__3-9-01__4-02 (1)
			3-23-01__3-9-01__4-02 (2)
			3-23-01__3-10-01__1-01
			3-23-01__3-16-01__3-02 (1)
			3-23-01__3-16-01__3-02 (2)
			3-23-01__3-16-01__4-01
			3-23-01__3-16-01__4-02
			3-23-01__3-22-01__1-01

			3-23-01__3-OR15-3__4-02<br>3-23-01__5-5-01__1-01<br>3-23-01__5-24-01__4-02<br>3-23-01__7-27-01__1-01 (1)<br>3-23-01__7-27-01__1-01 (2)<br>3-48-01__3-16-01__1-01<br>3-53-01__3-16-01__1-01
Pt2	2	T	3-48-01__3-16-01__4-01<br>3-48-01__3-16-01__6-02
		NT	3-23-01__3-16-01__6-02<br>3-48-01__2-21-01__6-02<br>3-48-01__2-OF15-2__4-02 (1)<br>3-48-01__2-OF15-2__4-02 (2)<br>3-48-01__2-OF15-2__6-02 (1)<br>3-48-01__2-OF15-2__6-02 (2)<br>3-48-01__3-3-01__6-02<br>3-48-01__3-9-01__6-02<br>3-48-01__3-10-01__6-03<br>3-48-01__3-16-01__4-01
	1	T	3-48-01__3-16-01__6-02
		NT	3-23-01__3-16-01__6-02<br>3-48-01__2-2-01__6-02<br>3-48-01__2-OF15-2__6-02<br>3-48-01__3-9-01__6-02<br>3-48-01__3-22-01__6-02<br>3-48-01__5-12-01__6-02<br>3-74-01__3-16-01__6-02
Pt3	2	T	3-23-01__3-10-01__4-01 (1)<br>3-23-01__3-10-01__4-01 (2)
		NT	3-23-01__0-IR-01C__4-01 (1)<br>3-23-01__0-IR-01C__4-01 (2)<br>3-23-01__0-IR-01C__4-01 (3)<br>3-23-01__2-2-01__4-01<br>3-23-01__2-IR2-01C__4-01<br>3-23-01__3-10-01__2-01<br>3-23-01__3-10-01__3-01<br>3-23-01__3-10-01__5-01<br>3-23-01__3-10-01__5-02<br>3-23-01__3-10-01__6-02<br>3-23-01__3-16-01__4-01<br>3-23-01__3-22-01__4-01<br>3-23-01__3-OR15-3__4-01
	1	T	3-23-01__0-IR-01C__2-01 (1)<br>3-23-01__0-IR-01C__2-01 (2)
		NT	3-23-01__2-2-01__2-01<br>3-23-01__3-3-01__2-01<br>3-23-01__3-22-01__2-01<br>3-23-01__5-12-01__2-01

*This Table lists the SoDA output, before manual examination. When there were two or more clones with the same V(D)J segments (but different in CDR3), they are marked with numbers – (1), (2), etc.

Table S5: Significance of differences in mutation distributions across different patients (χ^2 test)

Comparison	p-value (χ^2 test)
Pt1 tumor Vs non-tumor	**9*E-08**
Pt2 tumor Vs non-tumor	**4.3*E-4**
Pt3 tumor Vs non-tumor	**4*E-15**
Pt1 tumor Vs Pt2 tumor	**0.004**
Pt1 tumor Vs Pt3 tumor	**0.02**
Pt2 tumor Vs Pt3 tumor	0.39

<u>Table S6</u>: Significance of differences in mutation distributions across the different samples from the same patient (χ^2 test)

Patient No.	Comparison	p-value (χ^2 test)
1	Sample 3 tumor Vs Sample 2 tumor	0.88
	Sample 3 tumor Vs Sample 1 tumor	0.845
	Sample 3 tumor Vs non-tumor	**0.014**
	Sample 2 tumor Vs non-tumor	**1.5*E-05**
	Sample 1 tumor Vs non-tumor	**1.5*E-04**
2	Sample 1 tumor Vs Sample 2 tumor	**0.024**
	Sample 2 tumor Vs non-tumor	**0.022**
	Sample 1 tumor Vs non-tumor	0.10
3	Sample 1 tumor Vs Sample 2 tumor	**0.046**
	Sample 2 tumor Vs non-tumor	**7.7E-17**
	Sample 1 tumor Vs non-tumor	**0.04**

<u>Table S7</u>: Numbers of transition and transversion mutations in tumor and non-tumor clones across all FL patients, and in normal GCs

Sample		Number of mutations		p-value- (χ^2 test)
		Transitions	Transversions	
Pt1	T	417	504	0.58
	NT	491	531	
Pt2	T	526	489	0.08
	NT	214	141	
Pt3	T	567	790	0.60
	NT	230	356	
FL-S		1496	1117	
Normal GCs		367	289	

<u>Table S8</u>: Numbers of transition and transversion mutations in tumor and non-tumor clones across each sample from FL-HTS patients

Patient and sample			Number of mutations		p-value- (χ^2 test)
			Transitions	Transversions	
Pt1	Sample 3	T	109	154	0.051
		NT	279	266	
	Sample 2	T	80	147	**0.0014**
		NT	191	182	
	Sample 1	T	164	204	0.25
		NT	367	362	
Pt2	Sample 2	T	120	125	**0.001**
		NT	163	89	
	Sample 1	T	350	267	0.22
		NT	114	68	
Pt3	Sample 2	T	144	294	0.17
		NT	175	267	
	Sample 1	T	385	427	0.08
		NT	68	107	

Table S9: Numbers of positions in AID targeting motifs with significant differences between tumor and non-tumor clones in each sample

Patient 1			Patient 2		Patient 3	
Sample 3 T-NT	Sample 2 T-NT	Sample 1 T-NT	Sample 2 T-NT	Sample 1 T-NT	Sample 2 T-NT	Sample 1 T-NT
44	49	55	87	103	87	98

Table S10: Numbers of positions in AID targeting motifs with significant differences between different tumors in each patient

Patient 1		Patient 2	Patient 3
Sample 3 T-sample 2 T	Sample 3 T-sample 1 T	Sample 2 T-sample 1 T	Sample 2 T-sample 1 T
21	23	40	47

Table S11: Numbers of mutations in AID targeting motifs around each nucleotide, in tumor and non-tumor clones from FL patients and normal GCs

Sample		Number of mutations in motif			
		A	C	G	T
Pt1	T	126	106	64	54
	NT	161	111	143	46
Pt2	T	100	60	101	117
	NT	35	48	64	38
Pt3	T	136	102	142	91
	NT	84	28	69	51
FL-S		351	272	303	193
Normal GCs		77	69	81	65

Table S12: Numbers of mutations in AID targeting motifs around each nucleotide, in tumor and non-tumor clones in each sample from the FL-HTS patients

Patient and sample			Number of mutations in motif			
			A	C	G	T
Pt1	Sample 3	T	31	23	24	12
		NT	88	66	94	18
	Sample 2	T	23	21	15	8
		NT	57	50	82	4
	Sample 1	T	42	41	27	15
		NT	111	84	118	19
Pt2	Sample 2	T	33	19	20	23
		NT	26	41	45	21
	Sample 1	T	41	32	40	74
		NT	19	22	30	24
Pt3	Sample 2	T	39	42	33	25
		NT	73	20	52	41
	Sample 1	T	67	61	79	53
		NT	17	9	21	13

Table S13: Significance of the differences in percentages of the number of PGS and potential AGS in GL (χ^2 test)*

Patient / Sample	Vs. patient / sample	p-value (χ^2 test)
1 (samples 3+2)	2	**0.0013**
1 (samples 2+1)	2	**0.0013**
1 (samples 3+1)	2	**0.0017**
1 (samples 3+2)	1	0.28
1 (samples 2+1)	1	0.23
1 (samples 3+1)	1	0.27
2	3	**0.0008**
1 Tumor (samples 3+2)	2 Tumor	**0.0017**
1 Tumor (samples 2+1)	2 Tumor	**0.0017**
1 Tumor (samples 3+1)	2 Tumor	**0.0017**
1 Tumor (samples 3+2)	3 Tumor	0.375
1 Tumor (samples 2+1)	3 Tumor	0.375
1 Tumor (samples 3+1)	3 Tumor	0.375
2 Tumor	3 Tumor	**0.008**

*As Patients 2 and 3 had two samples each and patient 1 had three samples, we performed the χ^2 test for patients 2 and 3 twice, each time with each couple of samples from patient 1.
